# Unraveling the sugar code: the role of microbial extracellular glycans in plant–microbe interactions

**DOI:** 10.1093/jxb/eraa414

**Published:** 2020-09-15

**Authors:** Alan Wanke, Milena Malisic, Stephan Wawra, Alga Zuccaro

**Affiliations:** 1 University of Cologne, Cluster of Excellence on Plant Sciences (CEPLAS), Institute for Plant Sciences, Cologne, Germany; 2 Max Planck Institute for Plant Breeding Research, Cologne, Germany; 3 Heinrich-Heine-Universität Düseldorf, Germany

**Keywords:** Cell wall, chitin, extracellular polysaccharides, glucan, immunity, matrix, microbes, symbiosis

## Abstract

To defend against microbial invaders but also to establish symbiotic programs, plants need to detect the presence of microbes through the perception of molecular signatures characteristic of a whole class of microbes. Among these molecular signatures, extracellular glycans represent a structurally complex and diverse group of biomolecules that has a pivotal role in the molecular dialog between plants and microbes. Secreted glycans and glycoconjugates such as symbiotic lipochitooligosaccharides or immunosuppressive cyclic β-glucans act as microbial messengers that prepare the ground for host colonization. On the other hand, microbial cell surface glycans are important indicators of microbial presence. They are conserved structures normally exposed and thus accessible for plant hydrolytic enzymes and cell surface receptor proteins. While the immunogenic potential of bacterial cell surface glycoconjugates such as lipopolysaccharides and peptidoglycan has been intensively studied in the past years, perception of cell surface glycans from filamentous microbes such as fungi or oomycetes is still largely unexplored. To date, only few studies have focused on the role of fungal-derived cell surface glycans other than chitin, highlighting a knowledge gap that needs to be addressed. The objective of this review is to give an overview on the biological functions and perception of microbial extracellular glycans, primarily focusing on their recognition and their contribution to plant–microbe interactions.

## Teaser

This review highlights the structural diversity of microbial glycans and glycoconjugates, and focuses on their contribution to communication processes between plants and microbes.

## Introduction

Plants are immobile organisms that constantly sense and integrate information from biotic and abiotic cues in order to adapt to a dynamic environment. Monitoring of the microbial surrounding can be achieved by perception of molecular patterns that are either microbe-derived compounds [including structural microbe-associated molecular patterns (MAMPs) and microbial effectors] or host-specific molecules released or modified upon microbial activities, also referred to as damage-associated molecular patterns (DAMPs). Pattern sensing occurs via extracellular, membrane-integral pattern recognition receptors (PRRs) or intracellular receptors such as nucleotide-binding domain leucine-rich repeat-containing (NLR) proteins (Dodds and Rathjen, 2010; Cook *et al*., 2015). Upon recognition of these cues, subsequent receptor activation leads to signaling cascades that enable the plant to readjust its own physiological status towards defense (including pattern-triggered immunity and effector-triggered immunity) or symbiotic (i.e. mutualistic) programs (Zipfel and Oldroyd, 2017). Common features of microbe-triggered signaling are transphosphorylation cascades, ionic fluxes and membrane depolarization, apoplastic production of reactive oxygen species (ROS), transcriptional reprogramming, phytohormone signaling, production of secondary metabolites, and initiation of developmental programs (Couto and Zipfel, 2016; Zipfel and Oldroyd, 2017). Furthermore, these plant responses also hold the potential to influence and reshape the microbial composition in above- and below-ground tissues (Xin *et al*., 2016; Hacquard *et al*., 2017; Teixeira *et al*., 2019; Chen *et al*., 2020). However, the mechanisms governing the processing and integration of information obtained from the remarkable number of signaling events into an outcome that is overall favorable for the plant remain largely unclear and are under intensive investigation.

The nature of molecular patterns acting as indicators of microbial presence is highly diverse. It covers the most important classes of biopolymers, such as proteins and peptides, fatty acids, nucleic acids, and purine derivatives, as well as glycan-based substances (Gallucci and Maffei, 2017; Saijo *et al*., 2018; Nizam *et al*., 2019; Albert *et al*., 2020). Plants can sense an extremely wide spectrum of glycan substrates consisting of homo- and heteromeric polysaccharides and glycoconjugates. This includes endogenous sugar metabolites derived from carbon assimilation, plant cell wall components released upon damage, microbial glycoconjugate messengers dedicated to plant–microbe communication, as well as fibrous cell surface glycans involved in microbial biofilm and cell wall formation (Bolouri Moghaddam and Van den Ende, 2013; Limpens *et al*., 2015; Fesel and Zuccaro, 2016). Cell surface glycans in particular represent conserved and abundant targets for the plant’s surveillance system (Fesel and Zuccaro, 2016). On the other hand, microbes have evolved a plethora of tools and strategies to evade plant recognition (Rocafort *et al*., 2020).

The widespread potential for communication mediated by glycans lies in their structural complexity. While many biological polymers (e.g. polypeptides) are of linear nature, glycans are often built from small repeating basic units of monosaccharides that assemble into different three-dimensional flexible structures with a myriad of possible linkage types and branching patterns (Guberman and Seeberger, 2019). Chain length [also referred to as degree of polymerization (DP)], chemical modifications, and supramolecular structures have been shown to constitute relevant characteristics for specific recognition by the host (Ménard *et al*., 2004; Takahasi *et al*., 2009; Kanagawa *et al*., 2011; Zipfel and Oldroyd, 2017; Wanke *et al*., 2020).

This review provides an overview of the most important microbial glycan-derived patterns that are either directly perceived by plants or indirectly manipulate plant responses. Based on the diverse portfolio of biological and biophysical properties of microbe-derived polysaccharides, we highlight their potential to transmit information on the microbial composition (bacteria, fungi, and oomycetes) surrounding plant tissues.

## Judging a microbe by its ‘cover’: cell surface glycans and glycan-binding proteins in plant–microbe interactions

**Fig. 1.**
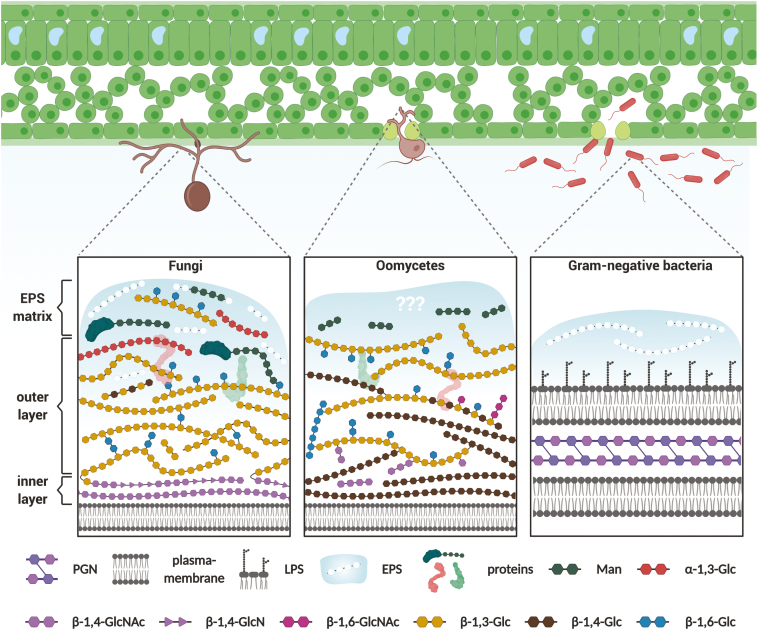
Schematic overview of microbial cell surface glycans. Recognition of microbe-derived cell wall polysaccharides represents an important mechanism by which plants surveil their microbial surrounding. Since cell wall structure and function are highly interlocked, core polysaccharides are conserved within different microbial groups. This scheme illustrates these core polysaccharides and their linkage types without representing exact quantitative proportions. Cell walls of filamentous fungi and oomycetes are network-like structures consisting of highly interconnected polysaccharide fibrils. In fungi, the inner cell wall layer consists of chitin (β-1,4-GlcNAc) and chitosan polymers (β-1,4-GlcN). It is covalently linked to the outer cell wall layer, which is mainly composed of β-1,3/1,6-glucans (β-1,3/1,6-Glc) with minor amounts of β-1,4-glucose (β-1,4-Glc). The outer layer is concealed by α-1,3-glucans (α-1,3-Glc) and mixed-linkage mannose (Man) polymers. Mannose polymers often occur as heterosaccharides with minor amounts of additional sugar types (e.g. rhamnose and galactose). A highly mobile, gel-like extracellular polysaccharide (EPS) matrix is loosely attached to the outer cell wall of many fungi. In contrast to fungi, no detailed studies on the cell wall architecture of oomycetes have been performed. Cross-linked cellulose (β-1,4-Glc) and β-1,3/1,6-glucans are major components of the inner part of oomycete cell walls. Chitin (β-1,4- and β-1,6-GlcNAc) only occurs in minute amounts; most of it is assumed to be connected to β-glucan polymers. The cellulose content is reduced in the external parts of the cell wall. Instead, branched β-glucans and mannose oligomers are present in that layer. To our knowledge, no detailed information on the architecture of an EPS matrix in oomycetes has been reported. Peptidoglycan (PGN) is a conserved part of bacterial cell walls present in Gram-positive and Gram-negative bacteria. The main heteropolysaccharides consist of alternating *N*-acetylglucosamine (GlcNAc) and *N*-acetylmuramic acid residues, which are interconnected through peptide chains. In Gram-negative bacteria, PGN is embedded between an inner (plasma membrane) and outer membrane layer. The outer membrane layer is decorated with lipid-linked polysaccharides, so-called lipopolysaccharides (LPSs). An amorphous matrix made of EPSs (e.g. xanthan and succinoglycan) encases many bacterial species. A more detailed overview on microbial cell surface glycans and their implications on plant–microbe interactions can be found in Supplementary Table S1.

Cell walls from bacteria and filamentous microbes are multilayered structures of fibrous homo- and heteropolysaccharides (Geoghegan *et al*., 2017; Gow *et al*., 2017) (Fig. 1). They act as mechanical support maintaining the cell turgor and determining cell shape and development (Shockman and Barrett, 1983; Money, 2008; Silhavy *et al*., 2010; Samalova *et al*., 2017). Although cell walls provide mechanical protection against adverse conditions and external aggressors, they can also be considered as an ‘Achilles’ heel’ in terms of their immunological potential (Supplementary Table S1 at *JXB* online). Upon colonization of the plant, they are one of the very first microbial components to be in contact with plant cells, either via direct physical contact or through the release of oligomers by a fleet of plant-secreted hydrolases (Rovenich *et al*., 2016). The basic functional principles of cell walls are comparable between different kingdoms; however, their molecular composition can be species specific (Coronado *et al*., 2007; Mélida *et al*., 2013; Gow *et al*., 2017). For that reason, plants evolved a broad surveillance system to recognize structurally different carbohydrate signatures (Albert *et al*., 2020). In the following sections we will describe the cell surface glycan compositions of bacteria and filamentous microbes (fungi and oomycetes) and highlight those glycan structures relevant for interaction with their hosts.

### Cell surface glycans in bacteria

Plants are able to perceive a complex array of glycan-based molecules that can be found in different layers of bacterial cell walls. Two major representatives are the glycoconjugates peptidoglycan (PGN) and lipopolysaccharide (LPS) (Fig. 1). PGN consists of a polymeric glycan-based backbone composed of alternating β-1,4-glycosidic-linked *N*-acetylglucosamine (GlcNAc) and *N*-acetylmuramic acid (MurNAc) residues (Vollmer *et al*., 2008). Oligopeptide bridges cross-link MurNAc residues of separate backbone polymers resulting in a multilayered structure (Vollmer *et al*., 2008). LPS, the second key building block, is composed of three covalently linked parts: Lipid A, the core oligosaccharide, and the O-antigen polysaccharide (Whitfield and Trent, 2014). Lipid A anchors LPS to the outer membrane via glucosamine-linked fatty acid chains. It is linked via 2-keto-3-desoxy-octonat (Kdo) to the core oligosaccharide which is composed of various sugar moieties. Lipid A and the core oligosaccharide together are also referred to as lipooligosaccharide and can be attached to a terminal O-antigen polysaccharide that is variable in size (Whitfield and Trent, 2014). While LPS is specific to Gram-negative bacteria, PGN exists in both Gram-positive and Gram-negative bacteria. Additionally, some bacterial species have capsular polysaccharides, which are tightly associated with the bacterial membrane and contain neutral or acidic polysaccharides (D’Haeze and Holsters, 2004). The composition of capsular polysaccharides varies among bacterial species and strains, but the presence of Kdo residues is commonly observed (Lopez-Baena *et al*., 2016). Moreover, a highly hydrated layer of polymeric substances, often referred to as extracellular matrix, surrounds bacterial cells. This layer includes proteins, extracellular DNA, and polysaccharides (Flemming and Wingender, 2010). Bacterial biofilm formation is supported by the extracellular matrix and creates a three-dimensional microenvironment where cell–cell communication takes place and abiotic and biotic stresses are tackled (Flemming and Wingender, 2010).

### The complex role of PGN and LPS in plant immunity

Bacterial cell wall glycans and glycoconjugates form conserved and unique structures in prokaryotes, which has driven the evolution of specialized surface-localized receptors in the hosts (Erbs and Newman, 2012; Ranf, 2016; Bacete *et al*., 2018). In *Arabidopsis thaliana* (hereafter Arabidopsis), PGN is perceived by a tripartite receptor complex consisting of lysin motif (LysM) domain receptor-like proteins LYM1 and LYM3 and the LysM receptor kinase CERK1 (Willmann *et al*., 2011). While LYM1 and LYM3 bind PGN, the transmembrane LysM receptor kinase CERK1 mediates intracellular signal transduction across the plasma membrane (Willmann *et al*., 2011). Similarly, PGN perception in rice is mediated through the respective orthologs LYP4, LYP6, and CERK1 (B. Liu *et al*., 2012; Ao *et al*., 2014). PGN signaling results in initiation of canonical immune responses such as medium alkalinization, cytoplasmic calcium influx, production of nitric oxide, phosphorylation of mitogen-activated protein kinases (MAPKs), camalexin accumulation, and induction of various defense-related genes (Gust *et al*., 2007; Erbs *et al*., 2008; Willmann *et al*., 2011; B. Liu *et al*., 2012; Ao *et al*., 2014; Liu *et al*., 2014). In addition to defense activation by complete PGN, there is evidence that specific PGN substructures trigger immune responses in Arabidopsis. Muropeptides derived from *Xanthomonas campestris* pathovar (pv.) *campestris* (*Xcc*) and *Agrobacterium tumefaciens* PGN preparations showed immunogenic potential in Arabidopsis (Erbs *et al*., 2008). Furthermore, glycan backbone structures from PGN preparations of *Staphylococcus aureus* also elicit immune responses in Arabidopsis (Gust *et al*., 2007). It has recently been shown that the Arabidopsis chitinase LYS1 can hydrolyze the *O*-glycosidic β-1,4-bond between MurNAc and GlcNAc residues generating soluble PGN products that mediate plant recognition (Liu *et al*., 2014). In line with PGN receptor mutants (Willmann *et al*., 2011), knockdown of LYS1 results in super-susceptibility to the phytopathogen *Pseudomonas syringae* pv. *tomato* DC300 (Willmann *et al*., 2011; Liu *et al*., 2014). However, overexpression of LYS1 also enhances susceptibility, indicating that quantitatively regulated PGN hydrolysis is important to generate immunogenic PGN fragments of a particular DP (Liu *et al*., 2014).

Upon recognition, LPSs can initiate classical immune responses such as cytosolic calcium influx, ROS generation, phosphorylation of MAPKs, and induction of several defense-related genes (Newman *et al*., 1995; Zeidler *et al*., 2004; Braun *et al*., 2005; Silipo *et al*., 2005; Madala *et al*., 2012; Desaki *et al*., 2018; Kutschera *et al*., 2019). Early studies promoted the notion that the Lipid A domain from LPSs triggers immune responses via the plant-specific bulb-type lectin kinase LORE in Arabidopsis (Ranf *et al*., 2015). However, later studies revealed that LPS and Lipid A preparations contained co-purified medium-chain 3'-OH-fatty acids (Kutschera *et al*., 2019). In fact, LORE mediates signal transduction upon recognition of medium-chain 3'-OH-fatty acids (fatty acid chain length: C8:0–C12:0). The LPS-bound fatty acid chains display the potential to be recognized by LORE, yet it has not been shown that they are hydrolytically released from LPS into the extracellular environment (Silipo *et al*., 2010; Ranf, 2016; Kutschera *et al*., 2019). Of note, two further LPS-binding proteins, LBR-1 and LBR-2, which share structural similarity with animal LPS-binding proteins (Iizasa *et al*., 2016), were identified in Arabidopsis (Iizasa *et al*., 2016, 2017). The impact on ROS generation and defense gene expression in *lbr* mutants upon LPS treatment suggests an active contribution of LBR1 and LBR2 to LPS recognition in Arabidopsis (Iizasa *et al*., 2016, 2017). A different perception mechanism was observed in rice, where the LysM receptor-like kinase CERK1 is required for LPS recognition (Desaki *et al*., 2018). Purified LPS induced ROS production and LPS-dependent transcriptional changes in wild-type-derived but not *cerk1* mutant-derived rice suspension-cultured cells (Desaki *et al*., 2018). However, whether CERK1 induces defense signaling through direct binding of LPS remains unknown. Additionally, the core oligosaccharides and O-antigen polysaccharides of LPS were reported to trigger plant defense responses (Bedini *et al*., 2005; Silipo *et al*., 2005; Madala *et al*., 2012; Kutschera *et al*., 2019). For instance, purified core oligosaccharides from *Xcc* are immunogenic in Arabidopsis (Silipo *et al*., 2005), and application of truncated LPS versions derived from *Xcc* mutants to tobacco cells suggests that the core oligosaccharide is important for LPS recognition (Braun *et al*., 2005; Silipo *et al*., 2005). The *P. aeruginosa* H4 strain is devoid of the O-antigen polysaccharide, lacks further sugar residues in the core oligosaccharide, and is not immunogenic, underpinning the importance of this region for immunorecognition in Arabidopsis (Kutschera *et al*., 2019). Transcriptional studies with purified LPS substructures from *Burkholderia cepacia* demonstrated defense gene induction in Arabidopsis by the entire polysaccharide (core oligosaccharide and O-antigen polysaccharide) in addition to LPS and Lipid A (Madala *et al*., 2012). This is supported by a study applying synthetic O-antigen polysaccharides (oligo-rhamnans) that induce defense-related gene expression in Arabidopsis in a DP-dependent manner, suggesting that the coiled structure is recognized by the plant (Bedini *et al*., 2005). Besides their immunogenic activity, the core oligosaccharide from *Xcc* and the synthetic O-antigen polysaccharides harbor the potential to suppress the hypersensitive response induced by bacterial infection (Bedini, 2005; Silipo, 2005). Furthermore, purified LPSs from phytopathogenic *Xylella fastidiosa* evoke defense responses independent of the presence of the O-antigen polysaccharide, while *in vivo* studies demonstrated the importance of this polysaccharide chain in shielding immunogenic surface structures and, thereby, delaying immunorecognition (Rapicavoli *et al*., 2018). Additionally, the rhizobial LPS from *Sinorhizobium fredii* HH103 is unique in its structure and was recently reported to have a role in the symbiotic establishment with its host legume *Macroptilium atropurpureum* and *Cajanus cajan* (Di Lorenzo *et al*., 2020). *Sinorhizobium fredii* HH103 core oligosaccharide differs from previously described rhizobial core oligosaccharides by the presence of a high number of hexuronic acids and a β-configured pseudaminic acid derivative. LPS from the *S. fredii* HH103 *rkpM* mutant lacks the pseudaminic acid derivative residues, leading to severely impaired nodule formation and symbiosis (Di Lorenzo *et al*., 2020). While previous studies already reported symbiotic impairments of the *S. fredii* HH103 *rpkM* strain with different legume hosts, now the responsible structural motifs were described (Margaret *et al*., 2012; Acosta-Jurado *et al*., 2016; Di Lorenzo *et al*., 2020). Although these data suggest recognition of LPS sugar modules, questions regarding the components mediating their recognition and signaling remain.

### Bacterial extracellular polysaccharides: a way to overcome immunity

During interaction with plants, both pathogenic and symbiotic bacteria secrete substantial amounts of extracellular polysaccharides (EPSs) into the apoplast. Several functions were attributed to bacterial EPSs such as sequestration of calcium ions, ROS scavenging, prevention of cellulose-mediated cell agglutination, tolerance to acidic pH, participation in biofilm formation, and host surface attachment (Laus *et al*., 2005; Aslam *et al*., 2008; Rinaudi and Gonzalez, 2009; Lehman and Long, 2013; Perez-Mendoza *et al*., 2015; Hawkins *et al*., 2017). The impact of EPSs on the plant host has been studied for several plant–bacterial systems. Xanthan, a polysaccharide consisting of repeating pentasaccharide units, is a key component of *Xcc* biofilms and suppresses plant immunity (Yun *et al*., 2006; Aslam *et al*., 2008). Its backbone is built by β-1,4-linked glucose residues and an additional trisaccharide side chain (mannose–glucuronic acid–mannose) at every second glucose unit (Yun *et al*., 2006). The internal mannose residues are substituted by an acetyl group, and half of the terminal mannose residues are substituted by either an acetyl- or a ketal-pyruvate residue (Yun *et al*., 2006). Due to its anionic nature, xanthan functions as a calcium chelator *in vitro* (Aslam *et al*., 2008). This capability is dramatically reduced in truncated xanthan, which is lacking the trisaccharide side chain with anionic modifications (Aslam *et al*., 2008). Arabidopsis pre-treatment with xanthan but not truncated xanthan prior to *Xcc* infection suppresses calcium influx-mediated defense responses such as ROS accumulation, expression of the defense gene *PATHOGENESIS-RELATED PROTEIN 1* (*PR1*), and formation of callose depositions (Yun *et al*., 2006; Aslam *et al*., 2008). Moreover, different types of EPSs such as xanthan from *Xcc*, amylovoran from *Erwinia amylovora*, alginate from *Pseudomonas aeruginosa*, or EPS from *Ralstonia solanacearum* and the symbiotic rhizobium *Sinorhizobium meliloti* hold the potential to reduce cytosolic calcium influx and ROS generation triggered by strong bacterial peptide elicitors such as flg22 and elf18 (Aslam *et al*., 2008). Since most of these EPSs have been shown to exhibit strong binding to calcium ions (Aslam *et al*., 2008), it is assumed that the immunosuppressive effect is conferred through apoplastic calcium chelation rather than through receptor-mediated signaling. This was also supported by the fact that xanthan did not compete with flg22 receptor binding (Aslam *et al*., 2008). Remarkably, a study in tomato revealed that EPSs from *R. solanacearum* might also trigger defense responses depending on the host plant (Milling *et al*., 2011). Supported by the finding that purified EPS from *R. solanacearum* specifically elicits salicylic acid (SA)-related defense gene expression in resistant but not in the susceptible tomato cultivars, this study suggests the emergence of an as yet unknown perception mechanism for *R. solanacearum* EPS in resistant tomato varieties (Milling *et al*., 2011).

In rhizobia–legume symbiosis (RLS), modification of bacterial EPS is commonly associated with impaired establishment of symbiosis on the level of nodule formation, infection thread initiation and elongation, bacteroid differentiation, and/or bacterial survival inside the nodules (Downie, 2010; Kelly *et al*., 2013; Arnold *et al*., 2018; Maillet *et al*., 2020). The mechanism governing this EPS-mediated compatibility was intensively studied for succinoglycan. Succinoglycan consists of a repeating octasaccharide monomer composed of a galactose residue and seven glucose residues that can carry additional succinyl, acetyl, and pyruvyl modifications. In interaction with the host *Medicago truncatula* (hereafter *Medicago*), *S. meliloti* succinoglycan- (EPS-I) deficient mutants or mutants lacking the succinyl residues fail to initiate infection thread formation and to invade the plant (Jones *et al*., 2008; Mendis *et al*., 2016; Maillet *et al*., 2020). Further, different early transcriptional responses to wild-type *S. meliloti* and a succinoglycan-deficient mutant (*exo*Y) were observed. While the presence of functional succinoglycan leads to active changes in root metabolism, the absence of succinoglycan induced a remarkably high number of plant defense genes (Jones *et al*., 2008). A mechanism for sensing compatible and incompatible EPSs was suggested for the *Mesorhizobium loti*–*Lotus japonicus* (hereafter *Lotus*) interaction. Screenings of different EPS-deficient *M. loti* R7A strains supported the notion that plants distinctly distinguish compatible EPSs from non-compatible EPSs in order to appropriately modulate defense responses and allow full infection thread development and bacterial release (Kelly *et al*., 2013). This model was supported upon identification of the *Lotus* LysM receptor kinase EPR3 that senses both native and truncated monomers of polymeric *M. loti* EPS in order to control bacterial progression through the plant’s epidermal cell layer (Kawaharada *et al*., 2015, 2017). The native *M. loti* EPS is *O*-acetylated and consists of an octasaccharide repeating monomer composed of glucose, galactose, glucuronic acid, and riburonic acid residues, whereas truncated EPS is a pentaglycoside lacking the terminal riburonic acid and glucuronic acid residues as well as another glucose unit (Kawaharada *et al*., 2015; Muszynski *et al*., 2016). Specificity between these two types of EPSs could be mediated by recruitment of distinct co-receptors (Kawaharada *et al*., 2015). Furthermore, a recent study proposes a more general role for EPR3 in surveying microbial compatibility due to its capability to bind to EPS monomers from different bacterial species (Wong *et al*., 2020). These findings represent a novel control mechanism for leguminous plants to differentiate between compatible and incompatible rhizobia to initiate consequent responses leading to negative or positive effects on bacterial progression (Kawaharada *et al*., 2017).

### Cell surface glycans in fungi

Fungal cell walls are mesh-like structures consisting of repeatedly branched glycan polymers and proteins (Fig. 1). They are not static constructions and continuously adjust according to cell type, environmental conditions, and lifestyle phases (Geoghegan *et al*., 2017; Gow *et al*., 2017). Yet, some basic structural elements are conserved and can be considered as fundamental features. Based on recent studies, three distinct layers of glycans can be found associated with the cell surface of most fungi: a stiff inner cell wall layer, a hydrated outer layer, and a highly mobile, gel-like EPS matrix, an often overlooked fungal glycan structure loosely attached to the cell wall outer layer (Kang *et al*., 2018; Wawra *et al*., 2019). The inner cell wall layer adjacent to the plasma membrane consists of a densely packed, hydrophobic layer of chitin (Osumi, 1998; Geoghegan *et al*., 2017). The chitin microfibrils are linear β-1,4-linked GlcNAc units, highly interconnected via hydrogen bonds, resulting in a rigid skeletal layer. Additional incorporation of melanin into this chitin layer increases resistance to oxidants and mechanical resilience as observed in the penetration structures (appressoria) of *Magnaporthe oryzae* (Chumley and Valent, 1990) and *Colletotrichum graminicola* (Ludwig *et al*., 2014). Some chitin fibrils are covalently linked to β-glucan chains, the predominant component of the outer cell wall layer of many fungi (~65–90% of polysaccharide content) (Bowman and Free, 2006). The β-glucans in this layer are mainly connected via β-1,3-linkages with regularly occurring β-1,6-side branches every 2–25 glucose units, thought to structurally interconnect the β-1,3-linked fibrils (Zekovic *et al*., 2005). In some cases, additional β-1,4-linked moieties and mixed-linkage β-1,3/1,4-glucose polymers can be observed (Fontaine *et al*., 2000; Pettolino *et al*., 2009; Kang *et al*., 2018). In contrast to chitin, β-glucans can aggregate into helical bundles or coils and form a branched network of non-crystalline fibrils that extends throughout the entire cell wall (Bluhm and Sarko, 1977; Bluhm *et al*., 1982; Osumi, 1998). On top of the β-glucan layer, interlinked α-1,3-glucans and/or mixed-linkage mannose polymers can be found (Geoghegan *et al*., 2017; Gow *et al*., 2017). These mannans can be attached to proteins (mannoproteins), galactose, and/or rhamnose moieties (Pettolino *et al*., 2009; Geoghegan *et al*., 2017; Gow *et al*., 2017; Mélida *et al*., 2018). In general, the inner and outer cell wall layer represent the fungal cell wall in the narrower sense of the term. Cytological analyses (for more information, see Box 1) have revealed the presence of an extensive gel-like EPS matrix surrounding hyphae of different human pathogenic and plant-colonizing fungi (Gow *et al*., 2017; Wawra *et al*., 2019). This highly mobile EPS matrix is not tightly bound to the cell wall and the bulk can be removed by washing. Similar to bacterial EPSs, the fungal EPS matrix is thought to be a loose scaffold for other macromolecules such as proteins, lipids, and extracellular DNA (Martins *et al*., 2010; Lee and Sheppard, 2016; Wawra *et al*., 2019). Compositional analyses of EPS matrices from different fungal species have identified glucose, mannose, galactose, rhamnose, xylose, and/or fucose as monosaccharidic building blocks (Mahapatra and Banerjee, 2013). More specifically, the EPS matrix from the saprotrophic fungus *Trametes versicolor* (Basidiomycota) was shown to contain a linear α-1,6-galactose backbone with mannose- and fucose-containing side chains (Scarpari *et al*., 2017). The EPS matrix of the root endophyte *Serendipita indica* (Basidiomycota) is composed of β-1,3/1,6-glucans (Wawra *et al*., 2019). Similarly, the plant pathogen *Botrytis cinerea* (Ascomycota) EPS matrix was shown to contain high molecular weight β-1,3/1,6-glucans (El Oirdi *et al*., 2011). While studies on the EPS matrix of plant-colonizing fungi are scarce, more information is available on the EPS matrix composition of human fungal pathogens. The EPS matrices from *Aspergillus fumigatus* and *Candida albicans* consist of complex galactans and mannans (Gow *et al*., 2017). Additionally, in *A. fumigatus*, a soluble galactosaminogalactan (GAG) made of a linear heteroglycan consisting of α-1,4-linked galactose and *N*-acetylgalactosamine was described (Gravelat *et al*., 2013). In the human pathogen *Cryptococcus neoformans*, the capsule (highly organized EPS matrix) contains glucuronoxylomannan (GXM) and glucuronoxylomanogalactan (GalXM). GXM is an α-1,3-mannose with β-1,2-linked xylose and glucuronic acid side chains, while GalXM consists of an α-1,6-galactan chain linked to galactomannan–xylose–glucoronic acid branches (Zaragoza *et al*., 2008; Denham *et al*., 2018; Decote-Ricardo *et al*., 2019). The large majority of studies report data originating from the analytics of whole-cell wall preparations without detailed knowledge of whether or not a mobile EPS matrix was present and how much of this matrix was still attached to the cell wall. It is therefore often difficult to speculate on the correct compositions of the EPS matrix or outer cell wall layer. Interestingly, the cell wall layers and EPS matrix seem to be synthesized by distinct pathways in *C. albicans*, accounting for the observed structural and functional differences (Taff *et al*., 2012; Mitchell *et al*., 2015).

**Box 1. Making the invisible visible: light microscopic approaches to study microbial cell walls**

Confocal laser scanning microscopy is a common tool used to assess microbial phenotypes during axenic growth and host colonization. The two major approaches for the visualization of microbes are live cell staining and staining after tissue fixation. Live cell microscopy allows the use of living samples, enabling dynamic imaging that can be used to track developments in real time and to obtain a better understanding of biological functions. This method requires easily applicable stains which do not interfere with the observed processes. Alternatively, fluorescently tagged marker proteins that localize to the region of interest can be employed. However, many organisms are not (easily) accessible to genetic engineering, especially when exhibiting an obligate biotrophic lifestyle. In these cases, exogenously applied probes are crucial tools to study the biology of these organisms. In contrast, fixation-based approaches lead to stable but inactive tissue, thus limiting the possibilities to study dynamic processes. Fixed material can be sectioned and is suitable for high-resolution light microscopy, electron microscopy, and electron microscopy tomography (Wang *et al*., 2019; Franken *et al*., 2020; Hansel *et al*., 2020). Yet, sample preparation can introduce artifacts through the fixation procedure and is often labor intensive. Optimization for the respective imaging technique and specific biological sample in particular can be a bottleneck (Bell and Safiejko-Mroczka, 1997; Wisse *et al*., 2010).

A key prerequisite for fluorescent labeling of microbes, particularly in host tissues or complex environments, is to target specific and unique microbial structures. Most of the fluorescent probes used to study microbes target cell walls or cell wall-associated components. These probes can be divided into four distinct groups: small chemical dyes; specific antibodies; fluorescent-labeled sugar-binding lectins; and engineered neolectins. The group of neolectins was reviewed in detail elsewhere and is therefore not covered here (Arnaud *et al*., 2013). Small chemical fluorescent dyes have been used for decades, combining the advantage of having high photostability and easy application. Common stains used for the visualization of filamentous microbes are the β-1,3/1,4-glucan-binding fluorescent dyes Calcofluor White/Fluorescent Brightener 28 (Leigh *et al*., 1985) and Congo Red (Darwish and Asfour, 2013). Despite the fact that both dyes target the same type of polysaccharide, they do not always localize to the same cell wall regions (Ursache *et al*., 2018). Aniline blue, another widely used glycan stain, interacts with β-1,3-glucans and is also capable of detecting chitosan (Evans *et al*., 1984; Becker *et al*., 2016). Aniline blue stains cell walls and has been used for decades to screen for EPS-producing microorganisms (Nakanishi *et al*., 1976). Yet, the exact nature of the interaction between these dyes and the respective glycans is still unknown. In addition, most of these small molecular fluorescent probes lack a clear specificity towards a single structure and, therefore, only function as proxies for the presence of a particular type of molecule or microbe.

In contrast to small fluorescent probes, monoclonal antibodies raised against specific glycans from microbial cell walls can display a high specificity and affinity towards the respective molecule used for immunization. However, many polysaccharides are generally far less immunogenic than proteins. While proteins generally display high internal heterogeneity and therefore carry various potential antigenic structures available for the animal immune system, large polysaccharides are often constructed of regular repeating flexible units. They often need to be conjugated to protein carriers to elicit an immune response (Cunto-Amesty *et al*., 2001; Cobb and Kasper, 2005; Zhou *et al*., 2009; Rydahl *et al*., 2017). Currently, there are only a limited number of polysaccharide-directed monoclonal antibodies available. Thus, the choices for detection of cell walls of filamentous microbes and associated structures are limited. Furthermore, the use of monoclonal antibodies in confocal laser scanning microscopy is mostly confined to fixed samples, and is time consuming and expensive.

**Fig. 2.**
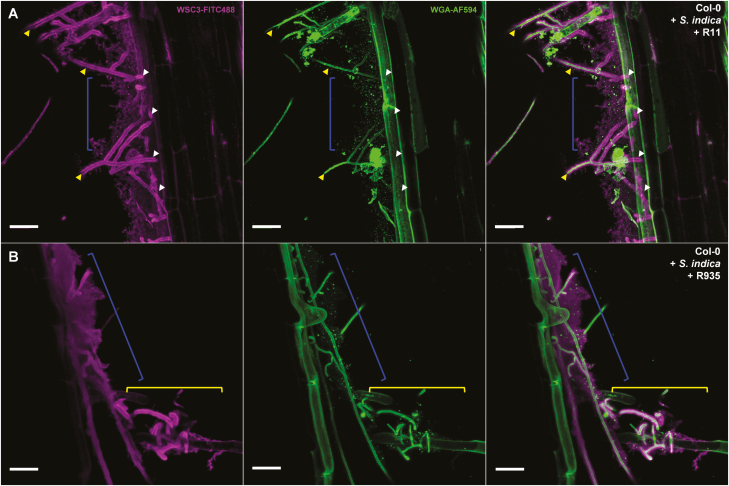
Live cell images of root-associated bacteria embedded in the fungal β-glucan matrix. Confocal laser scanning microscopy live stain images of an *A. thaliana* Col-0 root segment, 12 d post-colonization with the fungus *Serendipita indica* and root-associated bacteria R11 (*Bacillus* sp.) (A) or R935 (*Flavobacterium* sp.) (B) (Bai *et al*., 2015). The fungal cell wall and bacteria (dots) were stainable with WGA-AF594 (green pseudo-color). β-1,3-glucan was visualized using the FITC488-labeled lectin WSC3 (magenta pseudo-color) (Wawra *et al*., 2019). Fungal chitin was only detected in hyphae growing in the extracellular space (yellow arrowheads), whereas the β-glucan matrix was also detectable after the hyphae entered the root cortical cells (white arrowheads). Interestingly, the β-glucan architecture was very distinct in the presence of the individual bacterial strains (structures indicated by the blue brackets). While a fine structure in the glucan network was visible for *S. indica* in combination with R11, the combination with R935 resulted in an apparently extended, more dense, and amorphous looking β-glucan layer. In addition, in the presence of R935, the fungal β-glucan matrix around the *S. indica* hyphae appeared more diffuse and less compact compared with the matrix detected around more isolated growing hyphae (B, yellow brackets). It is currently unclear whether the additionally deposited β-glucan is produced by the bacteria themselves or whether their presence triggers enhanced production and secretion by the fungus. Scale bars=25 µm.

A promising alternative to monoclonal antibodies are lectins, which are glycan-binding proteins. In comparison with antibodies, these proteins have lower affinities towards their selective targets but have been shaped by evolution into molecules with highly specific binding. Compared with monoclonal antibodies, lectins are small and, if fluorophore tagged, easily applicable. The binding properties of lectins to their respective glycoligand are frequently defined by the geometric arrangement of their carbohydrate recognition domains. These domains as such often display rather low affinities for monosaccharides (Gabius, 1997). The biggest hurdle in using lectins as molecular probes lies in the determination of their glycan substrate specificity. The lack of commercially available and defined glycans for affinity screening largely limits the characterization of lectins binding to long and/or complex glycans. Therefore, most of the commercially available fluorescent lectins with defined affinities target small mono-, di-, or short oligosaccharides. A widely used lectin in the field of fungal biology is the GlcNAc-binding wheat germ agglutinin (WGA). However, GlcNAc is not only found in filamentous microbes, but also appears in the PGN layer of some bacteria or as building blocks in protein glycosylation (Quast and Lunemann, 2014; Lin *et al*., 2017). Thus, the ability of WGA to detect a short glycoligand reduces its structural specificity. To overcome this obstacle, the use of lectins that can distinctly bind long-chain glycoligands with low affinity for short-chain ligands of the same composition would be advantageous for the field. Recently, a fungal lectin exhibiting these characteristics was identified and characterized. The WSC3 protein from *S. indica* is a lectin composed of three WSC domains that binds to long-chain β*-*1,3-glucans with no measurable affinity for shorter β*-*1,3-linked glucose oligomers (<7) (Wawra *et al*., 2019). Optimization of the protein labeling procedure generated a very efficient probe, which is able to detect fungal EPS with very low background staining (Fig. 2). The only two described lectins able to detect fungal EPS are the β-1,3-glucan-binding WSC3 (Wawra *et al*., 2019) and the β-1,3/1,6-glucan-binding FGB1 (Wawra *et al*., 2016). A closer look into the microbial proteome associated with cell walls and EPSs could help to identify more lectins suitable as microscopic probes. Such probes would make a valuable contribution to the field, giving exciting insights into the structural composition of microbial cell walls and EPSs.

### Cell surface glycans in oomycetes—similar but different

Despite strong morphological and lifestyle similarities to fungi, oomycetes are a phylogenetically distinct group within the Stramenopiles clade, which also includes brown algae and diatoms (Adl *et al*., 2012; Beakes *et al*., 2012). The close evolutionary relationship with algae is reflected by the presence of cellulose (linear β-1,4-glucan) as a major cell wall component (33.6–51%) (Mélida *et al*., 2013). Comparative analyses of the cell wall composition from different oomycete species have demonstrated the presence of β-1,3/1,4/1,6-linked homo- and heteroglucans (85.6–95%). Furthermore, chitin as well as atypical β-1,6-linked GlcNAc can be found in cell wall preparations of representative species from the Saprolegniales order (i.e. the legume pathogen *Aphanomyces euteiches*) (Mélida *et al*., 2013). Additionally, the presence of cross-linkages of β-1,3-glucan with cellulose or GlcNAc/chitooligosaccharides was reported (Mélida *et al*., 2013; Nars *et al*., 2013). This association of chitin with other cell wall polymers accounts for the soluble, non-crystalline chitin fractions observed in the mycelial cell wall. In contrast to most true fungi, crystalline and non-crystalline chitin is thought to be distributed within the cell wall of oomycetes instead of being restricted to the inner layer (Badreddine *et al*., 2008). While chitin is reduced or absent from cell wall preparations of species from the Peronosporales order (e.g. Phytophthora genus), these cell walls additionally contain low amounts of mannan (6.7%) and glucuronic acid (4.7%) (Mélida *et al*., 2013). Few studies report on the presence of an EPS matrix secreted by encysting zoospores and germinating cysts in various oomycete species (Gubler and Hardham, 1988; Estrada-Garcia *et al*., 1990; Durso *et al*., 1993). In the germ tubes and appressoria of *Hyaloperonospora parasitica*, the causal agent of downy mildew in many crucifers, the EPS matrix consists of β-1,3-glucans, mannan, galactose, GlcNAc, and *N*-acetylgalactosamine (Carzaniga *et al*., 2001). More work is required to further address the presence and function of the EPS matrix during later stages of plant colonization.

### Hide and seek: chitin perception

In the last decades, chitin recognition in plants was intensively studied, leading to the elucidation of the main receptors and their downstream signaling pathways in several plant species (Sanchez-Vallet *et al*., 2015). A central player involved in chitin perception in different species is the LysM-domain receptor kinase CERK1. In Arabidopsis, recognition of chitooligomers (COs) with at least six GlcNAc units initiates the formation of ligand-induced heteromeric complexes between CERK1 and the LysM receptor kinases LYK4 and LYK5 (Miya *et al*., 2007; Wan *et al*., 2008; Petutschnig *et al*., 2010; Cao *et al*., 2014; Xue *et al*., 2019). Within this complex, LYK5 exhibits the highest affinity for COs, with a preference for oligomers having a DP of eight GlcNAc units (Cao *et al*., 2014). Binding to COs leads to the association of CERK1 with LYK5 and homodimerization of CERK1, which is needed for CERK1 autophosphorylation and further signal transduction (Petutschnig *et al*., 2010; T. Liu *et al*., 2012; Cao *et al*., 2014). LYK4 might act as a protein scaffold stabilizing the LYK5–CERK1 complex and thereby enhancing chitin-triggered responses (Xue *et al*., 2019). Furthermore, the weak, residual chitin response observed in *lyk5* mutants indicates that LYK4 together with CERK1 might also function as an additional receptor pair able to compensate LYK5 loss (Cao *et al*., 2014). In rice, chitin perception involves the CERK1 homolog and the receptor-like protein CEBiP, which is associated with the plasma membrane through a glycosylphosphatidylinositol (GPI) anchor (Kaku *et al*., 2006; Shimizu *et al*., 2010; Hayafune *et al*., 2014). Upon ligand binding, two CERK1 proteins associate with a pair of CEBiP receptors cooperatively binding to a single chitin molecule (Hayafune *et al*., 2014). Two additional rice GPI anchor receptor-like proteins, LYP4 and LYP6, also exhibit CERK1-mediated chitin binding affinity, which might function as accessory chitin perception machineries (B. Liu *et al*., 2012; Kouzai *et al*., 2014). Interestingly, CERK1-independent chitin sensing was reported within plasmodesmal membrane microdomains of Arabidopsis (Faulkner *et al*., 2013). Chitin-triggered plasmodesmata closure is controlled by LYM2, LYK4, and LYK5 (Faulkner *et al*., 2013; Cheval *et al*., 2020). LYM2 is a receptor-like protein with a GPI anchor which resides in plasmodesmal membranes (Faulkner *et al*., 2013). In response to chitin, LYM2 is suggested to oligomerize and act as a signaling platform for LYK4 and calcium-dependent protein kinases to initiate ROS production and callose synthesis (Cheval *et al*., 2020). These findings emphasize how a single elicitor initiates mechanistically independent responses in different membrane microdomains.

In addition to chitin-mediated activation of defense responses, several studies have shown the potential of COs (DP 4–8) to initiate nuclear calcium oscillations, a signaling hallmark during root symbiotic interactions with nitrogen-fixing rhizobia and arbuscular mycorrhizal fungi (Genre *et al*., 2013; Sun *et al*., 2015; Feng *et al*., 2019; He *et al*., 2019).

To respond to the widespread capacity of plants to detect microbial cell wall components, evolution has driven the development of a multilayered potpourri of microbial strategies to evade unintended recognition by plant receptors (Rovenich *et al*., 2016). Commonly observed mechanisms to avoid chitin-triggered immunity include inhibition or degradation of plant chitinases (Naumann, 2011; Jashni *et al*., 2015; Okmen *et al*., 2018) and replacement or partial conversion of chitin to chitosan, a deacetylated version of chitin with lower immunogenic potential in most plants (El Gueddari *et al*., 2002; Cord-Landwehr *et al*., 2016; Gao *et al*., 2019; Xu *et al*., 2020). Since plants lack the enzymatic toolset to degrade α-1,3-glucans, decoration of exposed cell wall regions of invading hyphae with these polysaccharides acts as a protective layer to circumvent chitin hydrolysis as was shown for *M. oryzae* (Fujikawa *et al*., 2012). A further layer of immunity evasion is conveyed by fungal chitin-binding effectors that are able to mask their own cell wall molecules (van den Burg *et al*., 2006; Marshall *et al*., 2011; Kombrink *et al*., 2017; Romero-Contreras *et al*., 2019; Zeng *et al*., 2020) or to sequester released COs (de Jonge *et al*., 2010; Marshall *et al*., 2011; Mentlak *et al*., 2012; Takahara *et al*., 2016; Romero-Contreras *et al*., 2019). Moreover, microbial effectors (Masachis *et al*., 2016; Fang *et al*., 2019; Irieda *et al*., 2019) or small RNAs (Jian and Liang, 2019) were shown to manipulate the host’s chitin perception and intracellular signaling machinery.

### The complexity behind perception of β-glucans from filamentous microbes

Despite the fact that β-glucans are predominant components of cell walls and EPS matrices in fungi and oomycetes, comparatively little is known about their perception by plants. Early studies could show that purified β-glucans from plant-colonizing fungi and oomycete cell walls are potent inducers of plant defense (Ayers *et al*., 1976b; Anderson, 1978). Pioneering work was performed on *Phytophthora sojae* culture filtrates and cell wall hydrolysates which identified β-1,3/1,6-glucans as potent elicitors of plant immunity resulting in phytoalexin accumulation (Ayers *et al*., 1976a, b; Keen and Yoshikawa, 1983; Okinaka *et al*., 1995). The smallest fragment with immunogenic capability was shown to be a purified β-glucan heptaglucoside composed of a β-1,6-linked backbone with two β-1,3-linked glucose side branches (Cheong *et al*., 1991; Sharp *et al*., 1984a, b). Later, a high-affinity β-glucan-binding protein (GBP) isolated from soybean root membrane fractions was proposed to act as part of a multimeric β-glucan receptor complex (Umemoto *et al*., 1997; Cheong *et al*., 1993; Fliegmann *et al*., 2004). GBP has a glucan-binding domain and β-1,3-glucanase activity (Umemoto *et al*., 1997; Fliegmann *et al*., 2004). Despite the lack of membrane targeting and secretion signals, GBP localizes to both the plasma membrane and apoplast (Umemoto *et al*., 1997). GBP inhibition via a specific antibody diminished β-glucan-elicited phytoalexin synthesis in soybean, indirectly validating its role in glucan perception (Umemoto *et al*., 1997). Although most plant species encode GBP homologs in their genomes, only legumes mount defense responses upon elicitation with the oomycete-derived β-glucan (Fliegmann *et al*., 2004). Whether this specificity is due to missing components of the receptor complex in other plant species or whether GBPs from non-leguminous plants bind to distinct β-glucan structures remains open. Alternatively, GBP activity could produce tailored β-glucan structures subsequently recognized by plant species-specific receptor systems. Other glucans such as short and unbranched β-1,3-linked glucose pentamers (laminaripentaose), hexamers (laminarihexaose), and laminarin, a β-1,3-glucan (DP 20–30) with β-1,6-linked side branches, can elicit immune responses in a wide range of plant species (Fesel and Zuccaro, 2016). Laminarin is a storage carbohydrate derived from marine brown algae which bears a remarkable resemblance to β-glucan structures present in many cell walls of filamentous microbes and, therefore, has often been used to investigate β-glucan-triggered immunity in plants and animals. Studies in monocot and dicot species have demonstrated that laminaripentaose (Klarzynski *et al*., 2000) and laminarihexaose (Mélida *et al*., 2018; Wanke *et al*., 2020) as well as laminarin (Klarzynski *et al*., 2000; Aziz *et al*., 2003; Ménard *et al*., 2004; Gauthier *et al*., 2014; Wawra *et al*., 2016; Wanke *et al*., 2020) activate immune responses such as calcium influx, ROS production, MAPK activation, medium alkalinization, expression of pathogenesis-related genes, phytoalexin synthesis, and phytohormone signaling. Moreover, laminarin is able to prime plant immunity and induces resistance to fungal plant pathogens and viruses in *Nicotiana tabacum* and *Vitis vinifera* (Aziz *et al*., 2003; Ménard *et al*., 2004; Gauthier *et al*., 2014). While the perception of laminarihexaose was shown to be mediated by CERK1 and an as yet unknown co-receptor in Arabidopsis (Mélida *et al*., 2018), long branched and unbranched β-glucans are recognized via a CERK1-independent pathway in *N. benthamiana* and rice (Wanke *et al*., 2020). Despite the fact that most plants are able to respond to β-glucans, it is striking that there are species-specific differences regarding the length, branching pattern, and presence of chemical modification (e.g. sulfation) of the perceived structures (Yamaguchi *et al*., 2000; Ménard *et al*., 2004; Wanke *et al*., 2020). The degree of variation in β-glucan recognition reflects the high structural diversity of microbial cell wall architectures between species. This implies that while chitin acts as a more general elicitor of plant immunity, β-glucan cues could allow for a rather specialized discrimination between different microbial groups.

A novel, chimeric type of elicitor was identified in glucanase- and chitinase-treated cell wall preparations of *A. euteiches* (Nars *et al*., 2013). This group of chito-glucans consists of a β-1,3/1,4-mixed-linkage glucan backbone with β-1,6-linked GlcNAc branches and is able to induce defense gene expression and nuclear calcium oscillation in *Medicago*. In contrast to lipochitooligosaccharides, chito-glucan-mediated nuclear calcium oscillations are not dependent either on the receptor kinase NFP or on the common symbiosis signaling pathway (Nars *et al*., 2013).

Cell wall β-glucans are crucial for microbial development and the formation of infection structures, but at the same time they pose the risk of disclosing the microbe’s presence to the plant. To successfully manage this trade-off, cell wall synthesis is highly synchronized to microbial development in a spatial and temporal manner. Investigations of glucan synthesis in the hemibiotrophic maize pathogen *C. graminicola* demonstrated that the glucan synthases GLS1, KRE5, and KRE6 participate in the production of β-1,3- and β-1,6-glucans. Invading biotrophic hyphae of this fungus coordinately down-regulate *GLS1, KRE5*, and *KRE6* expression, resulting in the formation of hyphae with little or no exposed β-1,3/1,6-glucans (Oliveira-Garcia and Deising, 2013, 2016). Overexpression of these glucan synthesis genes in biotrophic hyphae provokes callose production and defense gene expression. This highlights that dynamic remodeling of the cell wall architecture is an important strategy to subvert plant immunity (Oliveira-Garcia and Deising, 2013, 2016). Since plants have not been reported to possess β-1,6-glucanases, frequent branching patterns might spatially hinder host β-1,3-glucanases binding to their respective substrates and thus protect the microbe against cell wall hydrolysis (Nars *et al*., 2013). A further microbial strategy to safeguard its cell wall integrity is mediated via glucanase inhibitor proteins (Ham *et al*., 1997; Rose *et al*., 2002). These secreted proteins identified in the culture filtrate of *P. sojae* prevent cleavage of cell wall glucans by binding to host β-glucanases. The high-affinity complex formation is driven by a serine protease domain without a functional catalytic triad necessary for protein cleavage (Rose *et al*., 2002). More recently, the dual-function effector FGB1 from the beneficial root endophyte *Serendipita indica* was reported to mediate cell wall resistance to stress and to suppress β-glucan-triggered immunity in Arabidopsis and *N. benthamiana* (Wawra *et al*., 2016). FGB1 is a fungal lectin with a strong binding affinity for β-1,6-linked glucose residues (Wawra *et al*., 2016). This study draws attention to the fact that not only phytopathogenic but also beneficial fungi evolved mechanisms to circumvent recognition of their own cell wall components.

### The EPS of filamentous microbes: an emerging player in plant microbe-interaction?

Although several reports document the presence of highly mobile, gel-like EPS matrices surrounding the hyphae of plant-colonizing microbes, their function is largely unknown (Carzaniga *et al*., 2001; Wawra *et al*., 2019). Different types of EPS matrices were shown to contribute to host adhesion (Deising *et al*., 1992; Moloshok *et al*., 1993; Epstein and Nicholson, 1997; Doss, 1999; Carzaniga *et al*., 2001), act as an enzymatic scaffold (Nicholson and Moraes, 1980; Moloshok *et al*., 1993; Doss, 1999), or confer resistance to environmental stresses (Nicholson and Moraes, 1980) in different fungi and oomycetes. Still, little is known about their direct implications in interactions with plants. Analyses of β-glucan-containing EPS purified from culture supernatants of an endophytic *Fusarium* species (Li *et al*., 2014) and *B. cinerea* (El Oirdi *et al*., 2011) revealed activation of defense-related responses in their respective hosts. In *B. cinerea*, the EPS matrix induces accumulation of SA, an important player in the complex phytohormone signaling network regulating different defense pathways (El Oirdi *et al*., 2011). Whereas SA signaling often leads to host cell death and thereby restricts growth of biotrophic invaders, jasmonic acid (JA)-related defense generally accounts for resistance against necrotrophic pathogens (Glazebrook, 2005). Antagonistic crosstalk between these two phytohormones presents a fine-tuning mechanism to adapt immune responses according to different pathogenic lifestyles (Koornneef and Pieterse, 2008). This counteractive relationship between SA and JA is utilized by *B. cinerea* to increase disease progression in tomato through EPS-triggered SA accumulation and consequent suppression of JA-mediated pathways (El Oirdi *et al*., 2011).

A look into the body of literature on EPS matrices from *A. fumigatus* and *C. albicans* reveals that their EPSs can contribute to evasion of host immunity. In *A. fumigatus*, GAG polymers in the EPS matrix conceal immunogenic cell wall structures from recognition by host immune receptors (Gravelat *et al*., 2013). Furthermore, charges within the GAG polymers can protect the hyphae from antifungal peptides via electrostatic repulsion (Lee *et al*., 2015). Comparative genomic analyses identified the presence of a GAG biosynthetic gene cluster in plant-pathogenic fungi, indicating that GAG-like structures could play a role in plant pathogenesis (Lee *et al*., 2016). In *C. neoformans*, the GXM of its capsule was shown to confer resistance due to its function as a ROS scavenger (Zaragoza *et al*., 2008; Denham *et al*., 2018). Furthermore, capsular GXM and GalXM harbor immunomodulatory properties and contribute to host immune evasion (Decote-Ricardo *et al*., 2019). Similarly, the heterogalactan EPS from *Trametes versicolor* functions as a pro-antioxidant agent in mammalian cell lines, plants, and fungi (Scarpari *et al*., 2017). To what extent EPS matrices from plant-colonizing fungi or oomycetes can be considered fundamental virulence and symbiosis factors needs to be further addressed.

## Secreted microbial glycans: messengers of peace?

In addition to cell surface-attached glycans, microbes can secrete small and diffusible sugar oligomers and glycoconjugates that directly impact the outcome of plant–microbe interaction. While some function as signaling molecules to activate signaling cascades needed for the establishment of symbioses, others hold the potential to suppress immune responses in order to support successful plant colonization (Supplementary Table S1).

### The symbiont’s sweet words: small secreted glycoconjugates involved in the establishment of symbiotic interactions

Many plants form intimate associations with beneficial microbes. Among different forms of symbiotic interactions, arbuscular mycorrhiza (AM) and the RLS are two widespread representatives whose signaling pathways have been intensively studied for decades. While AM fungi improve the uptake of water and soil minerals in exchange for photoassimilates and lipids, rhizobia fix inert atmospheric nitrogen in specialized organs (nodules) functioning as an environmental niche for these bacteria. AM is genetically controlled by an ancient and highly conserved signaling pathway—the so-called common symbiosis signaling pathway—for recognition and initiation of symbiotic interactions (Oldroyd, 2013). In legumes, this pathway is required for the establishment of both AM and RLS. Several plant genes required for symbiosis have therefore been conserved throughout evolution in monocot and dicot plants (Genre and Russo, 2016). Since these bacterial and fungal symbionts share many structural patterns with pathogens, successful establishment of mutual interactions is dependent on the integration of stimuli obtained from both immunogenic and symbiotic signals. The main glycoconjugate messengers involved in the establishment of symbiosis are chitooligosacharides and lipochitooligosaccharides (LCOs), the latter also referred to as myc-LCOs (in AM) or nod-LCOs (in RLS) (Liang *et al*., 2014). Their perception activates pre-symbiotic signaling, gene expression changes. and developmental programs facilitating infection and the formation of symbiotic structures such as arbuscules and root nodules (Liang *et al*., 2014; Schmitz and Harrison, 2014). In general, LCOs are short, acylated COs (DP 3–5) with a variety of chemical modifications at their reducing and non-reducing termini. They are associated with a fatty acid at their non-reducing end and often carry further chemical modifications. The high diversity in chemical structure (backbone chain length, acetylation, sulfation, etc.) is assumed to mediate compatibility between host and microbe for specific establishment of symbiosis. Analogous to the perception of microbial cell wall components, the class of LysM motif receptor kinases is also involved in LCO signal recognition. In *Medicago*, nod-LCO perception is mediated via a heteromeric complex of the receptor-like kinases LYK3 and NFP (in *Lotus* NFR1 and NFR5) (Amor *et al*., 2003; Limpens *et al*., 2003; Madsen *et al*., 2003; Radutoiu *et al*., 2003; Arrighi *et al*., 2006). A previous study in *Lotus* suggested a two-step mechanism for establishment of nodulation in which nod-LCOs initiate symbiotic signaling and EPR3-driven EPS sensing acts as a subsequent quality control mechanism. Nod-LCO perception triggers the first step of symbiotic signal transduction, including intranuclear oscillatory calcium spiking at the root hair tip (Ehrhardt *et al*., 1996; Sieberer *et al*., 2009). Further, this induces EPR3 expression and initiates nodule organogenesis within the root cortex and bacterial infection through infection threads towards the nodule primordia (Gage, 2004). During these stages, iterative EPS recognition ensures colonization with compatible bacteria and promotes the progressing infection (Kawaharada *et al*., 2017). This collaborative recognition of nod-LCOs and rhizobial EPS presents a sophisticated way to repetitively control the advancing endosymbiosis without risking contamination with non-compatible bacteria. Of note, endophytic colonization of non-symbiotic bacteria (e.g. in mixed-colonized nodules) is also based on host-determined compatibility of their EPS (Zgadzaj *et al*., 2015). In addition to this well-established role of nod-LCOs, it was shown that nod-LCOs actively suppress defense signaling in different nodulating and non-nodulating species (Liang *et al*., 2013).

Initiation of AM is based on perception of myc-LCOs and COs, the two major classes of diffusible symbiotic signals found within fungal exudates. In *Medicago*, fungal myc-LCOs have been shown to trigger nuclear calcium spiking, alter gene expression patterns, and promote lateral root formation in an NFP-dependent manner (Maillet *et al*., 2011; Czaja *et al*., 2012; Sun *et al*., 2015). Unexpectedly, studies in *nfp* mutants in *Medicago* revealed that overall mycorrhization was not dependent on this receptor (Amor *et al*., 2003; Maillet *et al*., 2011). Notably, a recent study revealed that the LysM receptor-like kinase LYK10 confers binding to myc-LCO in non-leguminous plants. In contrast to the lacking phenotype in the Medicago *nfp* mutant, *lyk10* mutation impairs AM formation in tomato and petunia (Girardin *et al*., 2019). Since the family of LysM domain proteins is massively expanded in many plant species, it cannot be excluded that redundancy in the perception of myc-LCOs may cause the observed discrepancies.

Since perception of fungal COs also leads to oscillatory calcium fluxes, this glycoconjugate is assumed to play an important role in establishment of AM symbiosis (Genre *et al*., 2013). While short COs (DP 3–4) are often considered as symbiotic signals and long COs (DP 6–8) as defense patterns, recent work from Feng *et al*. (2019) demonstrates that COs independent of their length activate both defense and symbiotic signaling at the same time in *Medicago*. CO sensing involves the receptor kinases LYK9 (also CERK1) and LYR4 (Bozsoki *et al*., 2017). Combinatory application of both COs and LCOs was able to suppress immunity signaling in *Medicago*, leading to an overall symbiotic outcome (Feng *et al*., 2019). The integration of signaling cascades from different simultaneously perceived molecules forms an elegant mechanism supporting the plant to discriminate between detrimental or symbiotic microbes in order to respond with an adequate molecular program. Moreover, CO perception demonstrates that signaling mechanisms are entangled, often making it hard to classify signaling molecules within a dichotomous conception of immunity and symbiosis.

### Cellooligomers and cyclic β-glucans: secreted glycans in the extracellular space

Cellooligomers, β-1,4-linked glucose oligomers, can induce mild defense-like responses in plants. The strength of these responses in Arabidopsis and grapevine is strictly coupled to the DP (Aziz *et al*., 2007; Johnson *et al*., 2018). So far, these molecules were mainly considered as plant cell wall-derived molecules that are perceived by membrane-bound PRRs upon activity of pathogenic cell wall-degrading enzymes or during cell wall remodeling (Aziz *et al*., 2007; Souza *et al*., 2017). Only recently, cellotriose was discovered to be produced by the root endophyte *S. indica*. This trisaccharide triggers cytosolic calcium elevation, weak ROS induction, and membrane depolarization in Arabidopsis and induces the up-regulation of genes involved in plant defense (Johnson *et al*., 2018). Interestingly, a previous report on cellobiose-induced signaling in Arabidopsis demonstrated a ROS-independent host response including the activation of MAPKs and up-regulation of defense-related WRKY transcription factors (Souza *et al*., 2017). Cellotriose and cellobiose harbor the potential to synergistically increase host responses to chitin (Johnson *et al*., 2018) and flg22 when co-applied in Arabidopsis (Souza *et al*., 2017; Johnson *et al*., 2018). Additionally, pre-treatment of grapevine with cellooligomers of different DPs (DP 7–9) was shown to promote resistance against *B. cinerea* (Aziz *et al*., 2007), and cellobiose pre-treatment of Arabidopsis conferred enhanced resistance against the hemibiotrophic pathogen *P. syringae* pv. *tomato* DC3000 (Aziz *et al*., 2007; Souza *et al*., 2017). Yet, the components that mediate cellooligomer signaling remain unknown. In Arabidopsis, CERK1 and BAK1 receptor mutants remain sensitive to cellooligomers (Souza *et al*., 2017). Furthermore, cellooligomer-pre-treated grapevine plants do not present refractory behavior in their defense response to plant cell wall-derived oligogalacturonides, indicating a separate recognition mechanism (Aziz *et al*., 2007).

The fact that *S. indica* colonization of Arabidopsis (Vadassery *et al*., 2009) and cellotriose application (Johnson *et al*., 2018) both result in growth benefit for the plant suggests that cellotriose signaling might be involved in growth-promoting effects. Indeed, cellotriose treatment induced Arabidopsis genes involved in cell growth and root development, suggesting a genetically regulated mechanism for growth promotion (Vadassery *et al*., 2009; Johnson *et al*., 2018).

Cyclic β-glucans (CbGs) are produced by both pathogenic and symbiotic plant-colonizing bacteria and accumulate in the periplasmic and extracellular space (Breedveld and Miller, 1994). CbGs are involved in hypoosmotic adaption (Crespo-Rivas *et al*., 2009), motility (Breedveld and Miller, 1994; Bhagwat *et al*., 1996; Gay-Fraret *et al*., 2012), and stress protection (Javvadi *et al*., 2018). Bacterial mutants affected in CbG synthesis or periplasmic secretion form no or inefficient symbiosis or have reduced virulence, which emphasizes their relevance for plant–microbe interactions (Breedveld and Miller, 1994). CbGs can be separated into two groups based on the type of glycosidic linkage and DP. Bradyrhizobial β-1,3/1,6-linked cyclic glucans are built of 10–13 glucose molecules where the majority form a β-1,3-linked ring carrying branches of β-1,6-linked glucoses (Breedveld and Miller, 1994). In contrast, rhizobial cyclic glucans consist of 17–40 β-1,2-linked glucose moieties (Breedveld and Miller, 1994). Early evidence that CbGs can act as signaling molecules was derived from studies where the β-1,3/1,6-linked cyclic glucans from *Bradyrhizobium japonicum* were able to suppress the immuno-eliciting activity of β-1,3/1,6-glucans from the soybean pathogen *P. sojae* in a concentration-dependent manner (Mithöfer *et al*., 1996). The observation that both β-glucans bind to the same β-glucan-binding site in soybean roots suggests that the suppressive activity is mediated by competition. Bradyrhizobial CbGs were also shown to weakly induce production of the isoflavone daidzein, a known *Nod* gene inducer in *B. japonicum* which stimulates the production of bacterial LCOs (Mithöfer *et al*., 1996). Their impact on symbiotic interaction with soybean is dependent on the β-1,6-linked glucoses (Bhagwat *et al*., 1999). In contrast to bradyrhizobial CbGs, rhizobial CbGs (β-1,2-linked) from symbiotic *Rhizobium leguminosarium* and the phytopathogen *Agrobacterium tumefaciens* did not lead to the production of isoflavonoids (Miller *et al*., 1994). Furthermore, *S. meliloti* CbGs did not compete for the same receptor-binding site with the oomycete-derived β-1,3/1,6-glucans (Mithöfer *et al*., 1996).

CbGs from the leaf-colonizing phytopathogenic bacterium *Xcc* were shown to actively suppress local and systemic host defense responses. This type of CbG is structurally unique due to the presence of a single α-1,6-linked glycosyl residue among 15 β-1,2-linked glycosyl-residues (York, 1995). In *N. benthamiana*, CbGs from *Xcc* suppress *PR1* induction and, locally as well as systemically, callose deposition. Accordingly, pre-treatment of Arabidopsis and *N. benthamiana* with physiologically relevant concentrations of bacterial CbGs increased the susceptibility to *Xcc* in a systemic manner (Rigano *et al*., 2007). The *Xcc ndvB* mutant strain deficient in CbG production shows earlier and longer lasting *PR1* gene expression and enhanced callose deposition, resulting in lower virulence in Arabidopsis and *N. benthamiana* that could be restored by external application of CbGs (Rigano *et al*., 2007). Intriguingly, restoration of the symbiotic relationship between a CbG-deficient rhizobia strain and the legume host was not observed after external application of the respective CbGs, indicating a more complex role for rhizobial CbGs during plant–bacteria interaction (Dylan *et al*., 1990).

## Learning from the inconspicuous—a case study: the role of extracellular polysaccharides in the lichen symbiosis

An excellent example of the important role played by extracellular polysaccharides is given by the multipartite interactions occurring in lichens. Lichens are stable, long-term interactions between one or more multicellular fungi (mycobionts) and photosynthesizing single-celled algae or cyanobacteria (photobionts). These symbioses have repeatedly evolved complex three-dimensional architectures held together by hyphal cells (which represents up to 90% of the total biomass) and hydrophilic extracellular polymeric substances in the outermost layer. Below this hydrophilic layer, typically a water-repellent, gas-filled internal space is present where the photobiont is located, a prerequisite for efficient photosynthesis. Nutrients and water are diffusing from the EPS matrix into the fungal cell wall, and through the apoplast they reach the photobiont partners. Lichens and their photobionts are tolerant to desiccation and exhibit complete physiological recovery upon rehydration. Their water content is determined by environmental water availability and they are often subjected to alternating desiccation–rehydration cycles. When hydrated, the lichen thallus expands to many times its desiccated volume. The hydrophilic properties of the EPS matrix in the outmost layer account for this effect and determine the point at which surface tension is broken and water is taken up from the environment and retained (Spribille *et al*., 2020). Biochemical remodeling of the EPS matrix in desiccation-tolerant lichens is species-specific and seems to play a role in the response to changes in environmental water availability. Additionally, the EPS matrix serves as a surface-maximized depot for mineral nutrients and microbial-derived secondary metabolites. Because of its affinity for positively charged molecules, the EPS matrix plays an important role in heavy metal sequestration, preventing the uptake of toxic compounds into cells. This may explain the ability of certain pioneer lichens to colonize extremely polluted sites (Backor and Loppi, 2009). The EPS matrix also acts as substrate for colonization by bacteria and basidiomycetous yeasts commonly found in lichens. As in biofilms, the lichen EPS matrix functions as the main medium by which cell–cell communication between different microbial partners takes place (Spribille *et al*., 2020).

The EPS matrix in lichens can be considered as a multipartite structure. Its composition is not determined by a single organism but instead depends on the participating symbionts. Fungi are considered to be the major inhabitant contributing the bulk of secreted polysaccharides. Using histological stains, strongly acidic polysaccharides were visualized with distinguishable differences in anionic density between the fungal cell walls and the EPS matrix, suggesting that fungal sulfated polysaccharides and polyuronic acids are present in the matrix. Most of the reports have identified neutral β- and α-glucans and α-mannans with varying ratios of glucose, galactose, and mannan as the major components in whole lichens (Olafsdottir and Ingolfsdottir, 2001; Carbonero *et al*., 2002; Reis *et al*., 2002). The β-glucans are mainly represented by β-1,3- or β-1,3/1,4-glucans (lichenan), while the α-glucans are represented by the α-1,3/1,4-glucans isolichenan and nigeran, and less commonly by mixed-linkage α-1,4/1,6-glucan. Branched β-1,3/1,6-glucans and the β-1,6-glucan pustulan have also been reported in Lecanoromycetidae and Umbilicariomycetidae. These glucans are structurally similar to the core cell wall polysaccharides of fungi (Gow *et al*., 2017). In the few basidiomycete-based lichen symbioses, different cell wall components have been found with linear α-1,3-glucan (pseudonigeran). A comprehensive review of lichen EPSs is provided by Spribille *et al*. (2020). Despite the large amount of fungal biomass in lichens, other microbial partners may participate in the production of the extracellular polysaccharide matrix. Green algal symbionts are often found in the hydrophobin-lined internal chambers, generally not in direct contact with the matrix. However, recent reports have shown that algal partners can also excrete EPS, including medium to small sized uronic acid-containing polysaccharides and sulfated polysaccharides. The composition and amount of the secreted polysaccharides were shown to depend, at least in part, on abiotic stresses such as nitrogen, light and water availability, and presence of heavy metals (Gonzalez-Hourcade *et al*., 2020). Cyanobacterial symbionts, on the other hand, are typically embedded in a self-produced matrix. Bacterial and basidiomycetous yeast-derived EPSs in lichens are less studied but their potential functions should not be underestimated. Indeed the presence of the *nifH* gene involved in nitrogen fixation was demonstrated in several lichen-associated α-proteobacteria, γ-proteobacteria, actinobacteria, and firmicutes (Grube *et al*., 2009; Almendras *et al*., 2018). Prominent among the α-proteobacteria are the Rhizobiales with LAR1, a lichen-associated bacterial clade (Hodkinson and Lutzoni, 2009). It is therefore plausible to think that nitrogen-fixing bacteria represent a third important partner in the lichen symbiosis.

Understanding the structure and function of this highly organized EPS matrix in the extreme examples of lichen symbioses will help to shed light on the function of EPS matrices formed during multipartite interactions in the rhizoplane (Fig. 2) and their potential function in water, secondary metabolite, and mineral nutrient storage/utilization and immunity.

## Concluding remarks and perspective

The outcome of plant–microbe interactions is strictly governed by persistent communication processes between the involved partners. Due to the inherent structural and functional complexity of glycans and glycoconjugates, they represent an important group of messengers involved in this ongoing crosstalk. The high degree of specificity within this molecular dialog is influenced by various factors. Microbial cell walls, which are diverse networks of crystalline and soluble exopolysaccharides, often represent the starting point for recognition. While many of those structural motifs are highly conserved and can be recognized by a wide range of plants, perception of other unique structural motifs is limited to a few plant species. On the other hand, microbes secrete a fleet of molecules such as enzymes, effector proteins, and soluble glycans, in order to interfere with host recognition and the activation of defense responses. Collectively, the apoplastic encounter of plant and microbes gives rise to a multitude of signaling molecules, which need to be processed by both partners. Ancient endosymbioses such as AM and RLS highlight that collaborative receptor perception, continuous scrutiny, and integration of multiple signals is required to assess microbial compatibility and guide developmental reprogramming (Kawaharada *et al*., 2017). While our understanding of these signaling networks and their components is constantly increasing, many questions remain unanswered. The widespread use of heterogeneous substrates instead of pure and defined glycan structures is prompting the knowledge gap regarding receptors and downstream components for several glycan-based ligands (e.g. β-glucans and LPSs). Recent technological advances made in glycoscience, glycan synthesis, and structural biology will improve the quality of glycan substrates and help to revisit our understanding of how carbohydrate signatures contribute to host–microbe interactions (Weishaupt *et al*., 2017; Kang *et al*., 2018; Guberman and Seeberger, 2019; Reichhardt *et al*., 2019). Furthermore, not much attention has been paid to the impact of microbial EPS matrices surrounding plant organs. Although the composition and structural organization of EPS matrices differ between different microbial classes, functional convergence can be observed. Since EPS matrices of plant-colonizing bacteria and human-pathogenic fungi have been shown to be crucial determinants of host–microbe interactions, a similarly important function can be assumed for EPS matrices of filamentous microbes interacting with plants. Interestingly, bacteria and fungi commonly form mixed, interkingdom biofilms on plant tissues and in lichens (van Overbeek and Saikkonen, 2016; Guennoc *et al*., 2018). As observed in the latter, the EPS architecture within such multipartite consortia is jointly shaped and highly adapted to the demands of its inhabitants. Taking into account that each partner within collaborative microbial biofilms contributes its own set of enzymes involved in glycan synthesis and hydrolysis (Bamford *et al*., 2019), this could fuel the generation of unique community patterns. Therefore, it would be of great interest to understand whether such community-derived glycan signatures are relevant for microbiota assembly and plant recognition. The recent technological advances made in glycoscience, glycan synthesis, and structural biology will surely help to increase our understanding of these glycan structures and shed light on their contribution to host–microbe interactions.

## Supplementary data

The following supplementary data are available at *JXB* online.

Table S1. Overview of microbial glycans and glycoconjugate structures and their relevance for host–microbe interactions.

eraa414_suppl_Supplementary_Table_S1

## Data Availability

All data supporting the content of this study are available within the review and within its supplementary data published online.

## Abbreviations

AM: arbuscular mycorrhiza
CbG: cyclic β-glucan
CO: chitooligomer
DP: degree of polymerization
EPS: extracellular polysaccharide
GAG: galactosaminogalactan
GlcNAc: *N*-acetylglucosamine
GXM: glucuronoxylomannan
GalXM: glucuronoxylomanogalactan
GPI: glycosylphosphatidylinositol
Kdo: 2-keto-3-desoxy-octonat
LCO: lipochitooligosaccharide
LPS: lipopolysaccharide
LysM: lysin motif
MAPK: mitogen-activated protein kinase
MurNAc: *N*-acetylmuramic acid
PGN: peptidoglycan
PRR: pattern recognition receptor
pv: pathovar
RLS: rhizobium–legume symbiosis
ROS: reactive oxygen species
SA: salicylic acid
Xcc: *Xanthomonas campestris* pv. *campestris*
